# Diagnostic power of scleral spur length in primary open-angle glaucoma

**DOI:** 10.1007/s00417-020-04637-4

**Published:** 2020-03-07

**Authors:** Mu Li, Zhaoxia Luo, Xiaoqin Yan, Hong Zhang

**Affiliations:** 1 Department of Ophthalmology, Union Hospital, Tongji Medical College, Huazhong University of Science and Technology, Wuhan, 430022 China; 2 Department of Ophthalmology, Tongji Hospital, Tongji Medical College, Huazhong University of Science and Technology, Wuhan, 430030 China

**Keywords:** Scleral spur length, Schlemm’s canal, Primary open-angle glaucoma, Swept-source optical coherence tomography, Receiver operating characteristic curve

## Abstract

**Purpose:**

To investigate the diagnostic capability of scleral spur length in discriminating eyes with primary open-angle glaucoma (POAG) from healthy eyes.

**Methods:**

Seventy-eight eyes of 78 patients with POAG and 93 eyes of 93 age-, sex- and axial length-matched healthy subjects were included. The scleral spur length was measured using swept-source optical coherence tomography. Receiver operating characteristic (ROC) curves were derived based on the measurements.

**Results:**

The scleral spur length was significantly shorter in POAG eyes compared with healthy eyes (Method I, 164.91 ± 23.36 vs. 197.60 ± 25.32 μm; Method II, 145.15 ± 16.59 vs. 166.95 ± 19.31 μm; Method III, 162.33 ± 22.83 vs. 185.12 ± 23.58 μm, respectively; all *p* < 0.001). The areas under ROC curves were 0.841 (Method I), 0.810 (Method II), and 0.753 (Method III) for the scleral spur length. Moreover, Schlemm’s canal area was significantly associated with the scleral spur length (Method I) in both POAG (β = 0.027; *p* < 0.001) and healthy (β = 0.016; *p* = 0.009) groups.

**Conclusions:**

The scleral spur length had a good discriminating capability between POAG and healthy eyes, and it could be a novel biomarker for POAG evaluation clinically.

## Introduction

Primary open-angle glaucoma (POAG) is a leading cause of irreversible blindness worldwide [1]. The most important risk factor for POAG is the elevated intraocular pressure (IOP), and the reason for that is the increase in aqueous humor outflow resistance [2–4]. Previous studies have indicated that the majority (75%–90%) of aqueous outflow resistance is located in the region internal to Schlemm’s canal (SC), including the inner wall endothelium of SC, the basement membrane of SC, and the underlying juxtacanalicular connective tissue [5–7]. Thus, SC could be an important resistance locus in the aqueous humor outflow pathway [8, 9].

IOP, the autonomic nerve activity, and SC endothelial cell stiffness have been suggested to be able to affect the lumen size of SC [10–15]. Besides that, previous studies have reported that the scleral spur could also be a key factor of supporting the lumen size of SC. Via the posterior displacement of scleral spur, the force of ciliary muscle could stretch trabecular meshwork and the inner wall of SC, thus keeping SC lumen open [16–18]. When cutting the attachment between ciliary muscle and scleral spur off, the contraction of ciliary muscle induced by pilocarpine would be unable to affect the morphology of SC [19, 20]. In addition, Nesterov et al. found that the posterior SC, where received the most force from scleral spur, is wider than the anterior SC. This result also confirmed the importance of scleral spur on the morphology of SC [21]. Thus, in consideration of the close relationship between SC and scleral spur [16–21], and the close relationship between SC and aqueous humor outflow resistance [5–9], previous studies have suggested that short scleral spur could be engaged in the pathogenesis of POAG [16, 22, 23].

Moreover, using different measurement methods of scleral spur length, Swain et al. have reported that the mean scleral spur length was significantly shorter in POAG eyes compared with age-matched healthy eyes, indicating that a shorter scleral spur may be a risk factor in the progression of POAG because short scleral spur would be unable to support the lumen of SC [24]. This study gave us a significant clue of the association of scleral spur length with SC and POAG. However, this study was conducted using histological slides of cadaver eye in vitro*,* but not in real-time and in vivo, to observe and compare the scleral spur length of POAG and age-matched healthy eyes. As a noncontact and real-time method, the newly developed swept-source optical coherence tomography (SS-OCT) has a higher scan speed and a higher axial resolution, leading to a more detailed and clear in vivo observation of anterior chamber angle biometrics, including scleral spur and SC [25]. Accordingly, this study aimed to observe and compare the scleral spur length in both POAG and healthy eyes by SS-OCT in vivo and to investigate the capability of scleral spur length in discriminating glaucomatous eyes from healthy eyes.

## Materials and methods

This study was approved by the ethics committee of the Tongji Hospital, Huazhong University of Science and Technology, and adhered to the tenets of the Declaration of Helsinki. All subjects provided written informed consent prior to study participation.

### Subjects

Seventy-eight glaucomatous eyes of 78 patients with POAG (nobody included and tested used pilocarpine eyedrops) and 93 healthy eyes of 93 healthy subjects were recruited. All subjects received a comprehensive ophthalmic examination, including measurement of best-corrected visual acuity, refractive error (RE) (RT-2100, NIDEK CO.LTD, Gamagori, Japan), central corneal thickness (CCT) (corneal map, SS-OCT, CASIA SS-1000, Tomey Corp., Nagoya, Japan), axial length (AL) (IOL-Master, Carl Zeiss Meditec, Dublin, USA), slit-lamp examination (Haag-streit, Bern, Swiss), gonioscopy, fundus photography, IOP measurement, retinal nerve fiber layer thickness (RNFL) (spectral domain-OCT, Heidelberg Engineering GmbH, Heidelberg, Germany), and standard automated perimetry (Humphrey Field Analyzer, Carl Zeiss Meditec, Dublin, USA). Subjects were included in the POAG group if all of the following were true: (1) at least 18 years of age, (2) a glaucomatous-appearing optic nerve (rim thinning or focal notching), (3) RNFL defect was present, (4) glaucomatous visual field defects corresponding to optic nerve changes were present, and (5) normal anterior chamber depth with an open angle. Patients who had prior ocular surgeries were excluded from participation. Patients with systemic disease (e.g., hypertension, diabetic mellitus) were also excluded. Healthy subjects were included if all of the following were true: (1) at least 18 years of age, (2) IOP of ≤ 21 mmHg with no history of elevated IOP), (3) normal fundus, (4) no visible RNFL defect, (5) normal visual field, and (6) normal anterior chamber depth with an open angle. Potential control subjects were excluded from participation if they had a family history of glaucoma, a history of eye surgery, or systemic disease. One eye would be randomly selected from each recruited subject for SS-OCT examinations (for the measurement of scleral spur and SC).

### SS-OCT imaging acquisition and processing

SS-OCT (CASIA SS-1000; Tomey Corp., Nagoya, Japan) has scan speed of 30,000 A-scans/s and an axial resolution of less than 10 μm. The recruited subjects were imaged with the high-density (HD) scan [25]. The participants were instructed to open the eye wide during examination, and if necessary the examiner would lightly pull the participants’ upper or lower eyelid to expose the scan area adequately and avoid pressing the eye to ensure accurate measurements of SC and scleral spur. The superior, nasal, inferior, and temporal limbi were recorded separately after adjusting the fixture to the corresponding areas. The scans were performed three times and the best quality image was chosen for analysis.

### The measurements of scleral spur and SC parameters

**Fig. 1:**
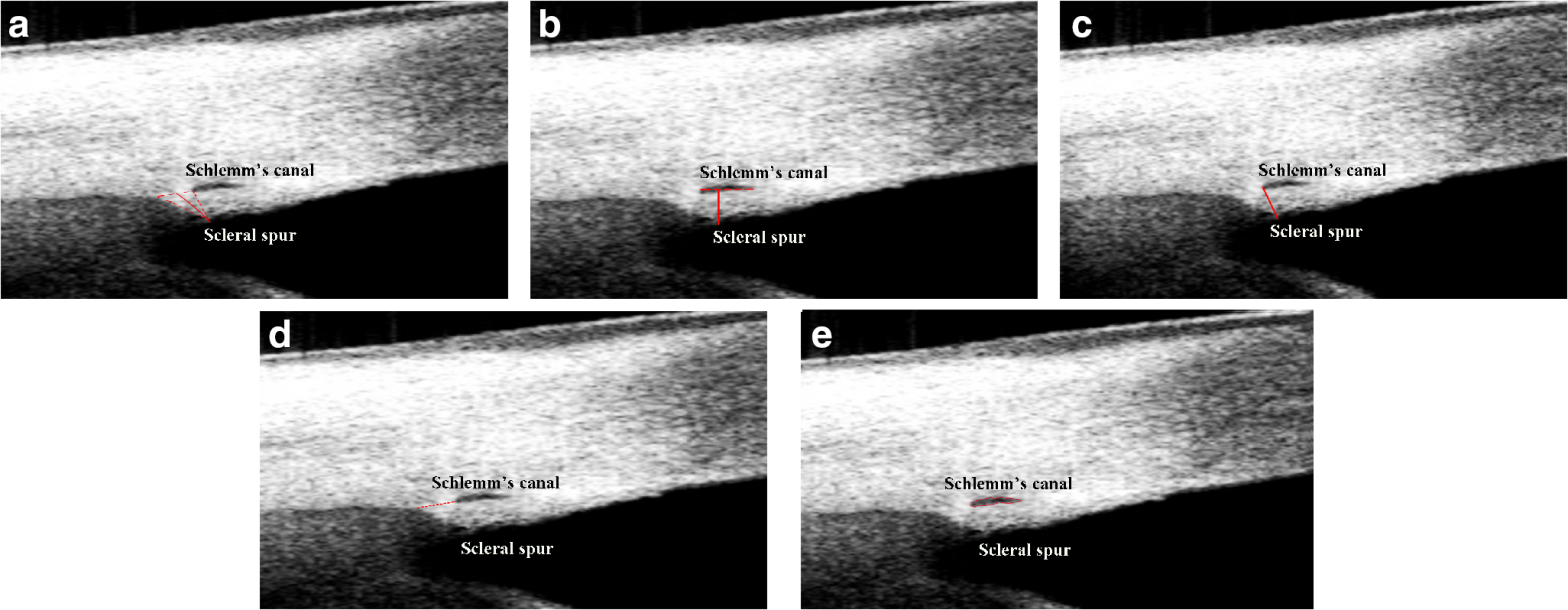
The measurement of scleral spur and Schlemm’s canal parameters. **a** The scleral spur measurement of Method I (Swain et al.): the measurement (the red solid line bisecting the width of the scleral spur at every point) was taken from the tip of the scleral spur to the middle of the red dotted line, which connects the anterior and posterior points where the sclera curves out to form the spur. **b** The scleral spur measurement of Method II (Nesterov and Batmanov): the measurement (the red solid line) was taken from the tip of the scleral spur, directly to the level of the posterior end of SC. **c** The scleral spur measurement of Method III (Moses and Arnzen): the measurement (the red solid line) was taken from the tip of the scleral spur to the level of the posterior end of SC, along the anterior side of the scleral spur. **d** The measurement of scleral spur opening width: the measurement (the red dotted line) was taken from the anterior point to the posterior point where the sclera curves out to form the spur. **e** The area of SC (the red curve) was manually drawn freehand based on the outline of SC

The scleral spur was a scleral protrusion at the anterior chamber angle and could be recognized in the SS-OCT image by following the boundary between the longitudinal fibers of ciliary muscle and the sclera until it reaches the anterior chamber [25]. SC was defined as a thin, black, lucent space in the HD image [26]. Optimum image contrast and magnification were subjectively defined in order to maximize the visualization of scleral spur and SC. The scleral spur length was measured in three different previously reported methods. Method I (Swain et al. [24]) (Fig. 1a): the red solid line (a curved line bisecting the width of the scleral spur at every point) represents scleral spur length, drawn from the tip of the scleral spur to the middle of the red dotted line, which connects the anterior and posterior points where the sclera curves out to form the spur (located near the posterior end of SC, to the point where the sclera begins again). Method II (Nesterov and Batmanov [21, 23, 24]) (Fig. 1b): the scleral spur length measurement (the red solid line) was taken from the tip of the scleral spur, directly to the level of the posterior end of SC. Method III (Moses and Arnzen [24, 27]) (Fig. 1c): the scleral spur length (the red solid line) was measured from the tip of the scleral spur to the level of the posterior end of SC, along the anterior side of the scleral spur. The width of scleral spur opening was measured as the length of the red dotted line, which connects the anterior and posterior points where the sclera curves out to form the spur (Fig. 1d). The area of SC was manually drawn freehand based on the outline of SC (the red curve) (Fig. 1e). All the measurements were achieved using the ImageJ software (National Institutes of Health, Bethesda, Maryland, USA) by two separate experienced operators (ML and ZL), who were masked to the subject information. Each eye had scleral spur and SC measurements taken in the superior, nasal, inferior, and temporal quadrants and the mean scleral spur and SC parameters (the average value of superior, nasal, inferior, and temporal quadrants measurements) were chosen for further analysis.

### Statistical analysis

All analyses were performed using R software version 3.4.3 (https://www.r-project.org). Data were presented as mean ± standard deviation (SD) where applicable. Differences in continuous variables between two groups were compared using independent *t* tests and Mann-Whitney *U* test, while categorical variables were compared using Chi-square test. The area under the receiver operating characteristic curve (AUC) was calculated to assess the capability of each testing parameter in differentiating glaucomatous eyes from healthy eyes, where AUCs of 1.0 and 0.5 represent perfect and chance discrimination, respectively [28]. The univariate and multivariate regression analysis was performed to quantify the associations of scleral spur length with demographic characteristics parameters. The interobserver and intraobserver reproducibility was assessed with the intraclass correlation coefficient (ICC). All tests were two-tailed and statistical significance was defined as *p* value < 0.05.

## Results

The demographic characteristics are shown in Table 1. There were no significant differences in age, sex, central corneal thickness (CCT), refractive error (RE), and axial length (AL) between POAG and healthy eyes (all *p* > 0.05). IOP was significantly higher (22.24 ± 7.42 vs. 15.03 ± 2.91 mmHg), and the mean deviation (MD) of visual field (− 11.43 ± 10.18 vs. − 1.20 ± 1.26 dB) and RNFL (71.38 ± 22.31 vs. 103.20 ± 10.94 μm) were significantly lower in POAG group compared with healthy group (all *p* < 0.001). Moreover, SC area of POAG group was significantly smaller than that of healthy group (4407.41 ± 430.25 vs. 4877.98 ± 434.33 μm^2^, *p* < 0.001).

**Table 1:** Demographic characteristics of study subjects

Characteristics	POAG eyes (*n* = 78)	Healthy eyes (*n* = 93)	*p*
Age (years)	42.65 ± 13.77	40.73 ± 12.57	0.346
Sex (male/female)	55/23	60/33	0.405
CCT (μm)	534.71 ± 31.64	533.13 ± 28.79	0.850
RE (D)	− 2.09 ± 2.89	− 1.67 ± 2.15	0.877
AL (mm)	24.37 ± 1.41	24.17 ± 1.25	0.777
IOP (mmHg)	22.24 ± 7.42	15.03 ± 2.91	< 0.001
MD (dB)	− 11.43 ± 10.18	− 1.20 ± 1.26	< 0.001
RNFL (μm)	71.38 ± 22.31	103.20 ± 10.94	< 0.001
SC area (μm^2^)	4407.41 ± 430.25	4877.98 ± 434.33	< 0.001

*POAG* primary open-angle glaucoma, *CCT* central corneal thickness, *RE* refractive error, *AL* axial length, *IOP* intraocular pressure, *MD* mean deviation, *RNFL* retinal nerve fiber thickness, *SC* Schlemm’s canal

### Comparison of scleral spur parameters between POAG and healthy groups

As Table 2 summarized, all the three measurement methods of scleral spur length showed that the scleral spur of POAG eyes was significantly shorter than that of healthy eyes (164.91 ± 23.36 vs. 197.60 ± 25.32 μm (Method I); 145.15 ± 16.59 vs. 166.95 ± 19.31 μm (Method II); 162.33 ± 22.83 vs. 185.12 ± 23.58 μm (Method III), all *p* < 0.001). In addition, the scleral spur opening width was also significantly narrower in POAG eyes compared with healthy eyes (135.01 ± 42.11 vs. 168.32 ± 42.36 μm, *p* < 0.001).

**Table 2:** Comparison of scleral spur parameters between healthy and POAG group

	POAG eyes (*n* = 78)	Healthy eyes (*n* = 93)	*p*
Scleral spur length (μm; Method I)	164.91 ± 23.36	197.60 ± 25.32	< 0.001
Scleral spur length (μm; Method II)	145.15 ± 16.59	166.95 ± 19.31	< 0.001
Scleral spur length (μm; Method III)	162.33 ± 22.83	185.12 ± 23.58	< 0.001
Scleral spur opening width (μm)	135.01 ± 42.11	168.32 ± 42.36	< 0.001

*POAG* primary open-angle glaucoma

**Fig. 2:**
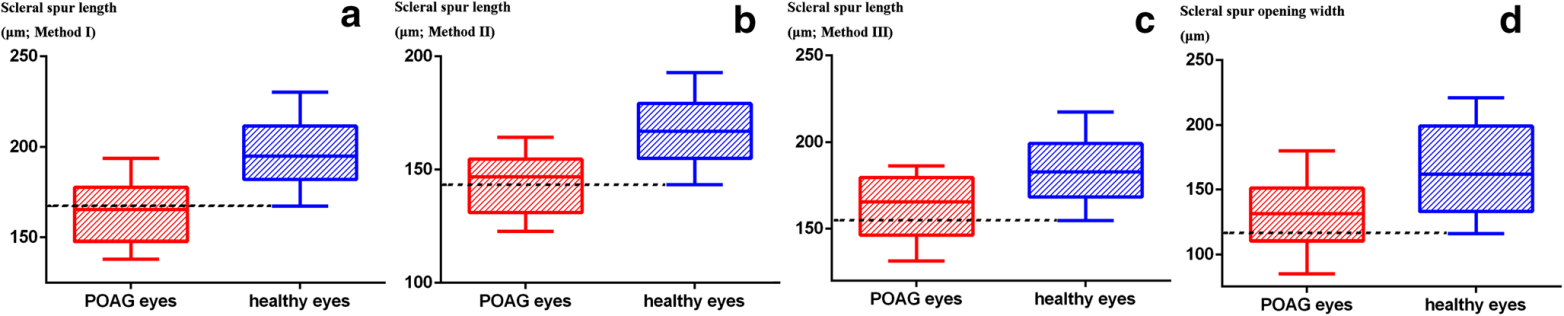
Box plots showing the distributions of scleral spur lengths (**a**–**c**) and scleral spur opening width **d** in both groups. Dashed lines indicated the lower 90th percentile values in healthy subjects for scleral spur length and scleral spur opening width

Figure 2 shows the distribution of scleral spur lengths and scleral spur opening width in both groups. The lower 90th percentile values of scleral spur lengths in healthy subjects were 167.49 μm (Method I), 143.72 μm (Method II), and 155.00 μm (Method III), respectively. The scleral spur lengths were smaller than the lower 90th percentile values in healthy subjects in 42 (55.3%) (Method I), 32 (41.0%) (Method II), and 28 (35.9%) (Method III) POAG patients, respectively. For scleral spur opening width, the lower 90th percentile value in healthy subjects was 116.42 μm, and the scleral spur opening width was smaller than the lower 90th percentile value in healthy subjects in 26 (33.3%) POAG patients.

**Fig. 3:**
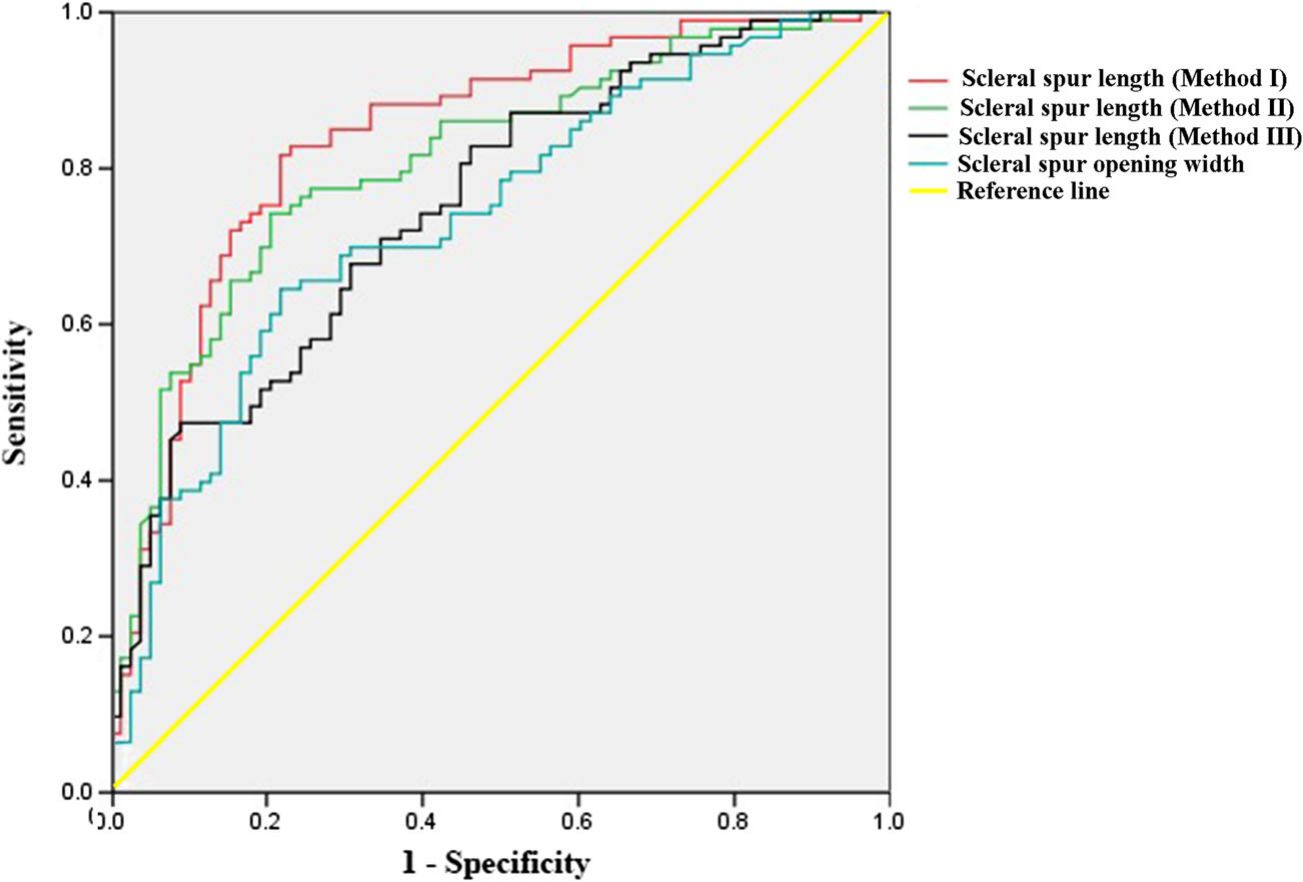
Receiver operating characteristic curves for scleral spur length and scleral spur opening width

The AUCs of scleral spur length and scleral spur opening width to discriminate POAG subjects from healthy subjects are shown in Fig. 3 and Table 3. The areas under those curves for the scleral spur length were 0.841 (95% confidence interval (CI) 0.780–0.902) (Method I), 0.810 (95%CI 0.746–0.875) (Method II), and 0.753 (95%CI 0.681–0.825) (Method III), respectively. The area under the curve for the scleral spur opening width was 0.737 (95%CI 0.662–0.811).

**Table 3:** Receiver operating characteristic (ROC) curves of scleral spur parameters between POAG and healthy groups

	AUC	*p*	Cut-off value	Sensitivity	Specificity
Scleral spur length (μm; Method I)	0.841	< 0.001	179.79 μm	0.782	0.817
Scleral spur length (μm; Method II)	0.810	< 0.001	155.69 μm	0.795	0.742
Scleral spur length (μm; Method III)	0.753	< 0.001	186.38 μm	0.910	0.473
Scleral spur opening width (μm)	0.737	< 0.001	151.70 μm	0.782	0.645

*AUC* area under receiver operating characteristic curve

In addition, the AUC value for scleral spur length (Method I) was significantly larger than that for scleral spur length (Method III) (*p* = 0.001, 95%CI 0.037–0.138) and that for the scleral spur opening width (*p* = 0.033, 95%CI 0.008–0.199) (Table 4).

**Table 4:** Comparison of the AUCs between scleral spur length (Method I) and scleral spur length (Method II, Method III), scleral spur opening width

	AUC	95% CI	*p*
Scleral spur length (μm; Method I)	0.841	0.780–0.902	
Scleral spur length (μm; Method II)	0.810	0.746–0.875	0.386^1^
Scleral spur length (μm; Method III)	0.753	0.681–0.825	0.001^2^
Scleral spur opening width (μm)	0.737	0.662–0.811	0.033^3^

*AUC* area under receiver operating characteristic curve, *CI* confidence interval

^1^*p* value between scleral spur length (Method I) and scleral spur length (Method II),^2^*p* value between scleral spur length (Method I) and scleral spur length (Method III),^3^*p* value between scleral spur length (Method I) and scleral spur opening width

### Univariate and multivariate regression between scleral spur length and demographic characteristic parameters

We chose the scleral spur length measurement of Method I, which had the largest AUC, to investigate the associations of scleral spur length with demographic characteristics parameters. The multivariate regression results showed that in both POAG and healthy groups, the SC area was significantly associated with scleral spur length (Method I) (β = 0.027 and 0.016, respectively; *p* < 0.001 and = 0.009, respectively). Besides that, no significant associations between scleral spur length (Method I) and age, sex, CCT, AL, RE, and IOP were found (all *p* > 0.05) (Table 5).

**Table 5:** Univariate and multivariate regression between scleral spur length and demographic characteristics parameters

	Scleral spur length of POAG eyes				Scleral spur length of healthy eyes			
	Univariate		Multivariate		Univariate		Multivariate	
	β[95%CI]	*p*	β[95%CI]	*p*	β[95%CI]	*p*	β[95%CI]	*p*
Age (years)	0.354 [− 0.019, 0.727]	0.066	0.010 [− 0.409, 0.428]	0.963	0.461 [0.059, 0.864]	0.027	0.183 [− 0.312, 0.677	0.471
Sex	9.798 [− 1.430, 21.026]	0.091	1.734 [− 8.790, 12.259]	0.748	5.812 [− 4.936, 16.560]	0.292	2.842 [− 8.583, 14.266]	0.627
CCT (μm)	− 0.075 [− 0.240, 0.091]	0.379	− 0.054 [− 0.203, 0.095]	0.476	− 0.001 [− 0.181, 0.180]	0.994	0.019 [− 0.174, 0.213]	0.846
RE (D)	1.404 [− 0.387, 3.195]	0.129	1.860 [− 0.929, 4.649]	0.195	2.372 [− 0.029, 4.773]	0.051	− 0.294 [− 5.236 4.647]	0.907
AL (mm)	− 2.304 [− 5.988, 1.380]	0.224	1.027 [− 4.858, 6.912]	0.733	− 4.896 [− 8.943,− 0.849]	0.020	− 4.511 [− 13.004, 3.983]	0.301
IOP (mmHg)	− 0.134 [− 0.841, 0.574]	0.712	− 0.025 [− 0.676, 0.627]	0.941	− 0.089 [− 1.879, 1.701]	0.923	0.073 [− 1.809, 1.954]	0.940
SC area (μm^2^)	0.027 [0.017, 0.038]	< 0.001	0.027 [0.015, 0.039]	< 0.001	0.016 [0.004, 0.027]	0.008	0.016 [0.004, 0.028	0.009

*POAG* primary open-angle glaucoma, *CCT* central corneal thickness, *RE* refractive error, *AL* axial length, *IOP* intraocular pressure, *SC* Schlemm’s canal, *CI* confidence interval

### Interobserver and intraobserver reproducibility of scleral spur and SC parameter measurements

The results of Table 6 showed that the interobserver and intraobserver reproducibility of this study was good. The ICC values of the measurements were at the range from 0.857 to 0.931 (interobserver) and from 0.868 to 0.940 (intraobserver).

**Table 6:** Interobserver and intraobserver reproducibility of scleral spur and SC parameter measurements

Interobserver	ICC	Difference	95%CI	
			Lower	Upper
SC area (μm^2^)	0.857	26.30	0.812	0.893
Scleral spur length (μm; Method I)	0.885	4.70	0.848	0.914
Scleral spur length (μm; Method II)	0.922	0.67	0.896	0.942
Scleral spur length (μm; Method III)	0.931	0.53	0.908	0.948
Scleral spur opening width (μm)	0.880	7.36	0.842	0.910
Intraobserver	ICC	Difference	95%CI	
			Lower	Upper
SC area (μm^2^)	0.879	73.02	0.840	0.909
Scleral spur length (μm; Method I)	0.898	4.32	0.864	0.923
Scleral spur length (μm; Method II)	0.921	0.89	0.895	0.941
Scleral spur length (μm; Method III)	0.940	0.43	0.919	0.955
Scleral spur opening width (μm)	0.868	8.04	0.825	0.901

*ICC* intraclass correlation coefficient, *CI* confidence interval, *SC* Schlemm’s canal

## Discussion

In this study, using SS-OCT, we investigated the scleral spur length in both POAG and healthy eyes and found that the scleral spur length of POAG eyes was significantly shorter than that of healthy eyes. The scleral spur length also showed a good diagnostic capability in discriminating POAG eyes from healthy eyes. Moreover, the SC area was significantly associated with the scleral spur length in both POAG and healthy groups.

The scleral spur is a wedge-shaped circular ridge protruding out of the inner sclera, from the corneoscleral portion of trabecular meshwork to the longitudinal fibers of the ciliary muscle [18]. The scleral spur contains circumferentially oriented elastic and collagenous fibers and spindle-shaped, circularly oriented contractile myofibroblast cells (scleral spur cells) [29, 30]. Among the fibers, the elastic fibers of the scleral spur are continuous posteriorly with the elastic fiber tendons of the longitudinal fibers of ciliary muscle and anteriorly with the elastic fibers in TM, along with the juxtacanalicular tissue underneath the SC inner wall endothelium [18, 29]. Thus, the scleral spur has long been supposed to be a supporting tissue for SC and has an impact on the aqueous humor outflow facility [24]. The posterior displacement of the scleral spur could transmit the force of ciliary body to the trabecular meshwork and SC, stretch them, and keep them open [16–18]. Using immersion-fixed enucleated eye tissue by histological method, the study of Swain et al. reported that the scleral spur of POAG eyes was significantly shorter than that of healthy eyes and the shorter scleral spur length could not provide required support for the patency of SC. In addition, the shorter scleral spur also has less ciliary muscle and trabecular meshwork attachment than normal eyes. When the ciliary muscle contracts and pulls the scleral spur of POAG eyes, it moves only a shorter distance posteriorly, opening fewer layer of meshwork beams and failing to support SC lumen. Thus, the shorter scleral spur of POAG eyes could compromise the “ciliary muscle-scleral spur-trabecular meshwork” network, which is important in maintaining the patency of SC. Based on this, Swain et al. speculated that individuals with a shorter scleral spur may be at a greater predisposition to POAG compared with the healthy counterparts with longer scleral spur [24].

Using SS-OCT, we measured the scleral spur length in POAG and healthy groups in this study. The scleral spur was made up of fibers [29], making it little affected by the application of anti-glaucoma drugs. Thus, although most of the POAG eyes included in this study were under anti-glaucoma drug treatment, the glaucomatous scleral spur length we measured here might not be affected by the anti-glaucoma drug and could be relatively comparable to its treatment-naïve status, ensuring the reality and validity of glaucomatous scleral spur length measurement and excluding the confounding effect of anti-glaucoma drugs in this study. Our results of in vivo scleral spur length measurements were similar to those of Swain et al. [24], showing that the scleral spur length of POAG eyes was significantly shorter than that of healthy eyes. In addition, given that a shorter scleral spur length could be a potential pathogenesis of POAG, we also performed ROC curves analysis to investigate the diagnostic capability of scleral spur length in discriminating POAG eyes from healthy eyes. The results showed a good discriminating capability (AUC value = 0.841), and it was comparable to the previously reported respective values of other diagnostic parameters for glaucoma (e.g., global peripapillary RNFL thickness has AUCs ranging from 0.87 to 0.94 [31–33] and optic nerve head has AUCs ranging from 0.77 to 0.97 [34–36]). The high AUC value of scleral spur length indicated that this parameter could be used as a novel biomarker for POAG evaluation clinically.

We have also performed multivariate regression equation to determine the association between SC area and scleral spur length, and the results showed that irrespective in POAG or in healthy group, the SC area was significantly associated with the scleral spur length; the longer scleral spur could better support SC, resulting in the larger SC area. This result confirmed the previous study conclusions, which indicated the important role of scleral spur in the patency of SC [16–18, 24]. However, no other significant associations between age, sex, AL, RE, CCT, IOP, and scleral spur length were found. The reason for the no significant association between IOP and scleral spur length could be: first, most of the study recruited POAG eyes were under anti-glaucoma drugs treatment. Thus, the posttreatment IOP was affected and controlled by drugs and might not be comparable to the actual SC and glaucoma status. Second, besides SC, which was closely associated with the scleral spur length, there were also other factors that could contribute to the glaucomatous IOP (e.g., the glaucomatous changes in biomechanics and structures of SC and trabecular meshwork, the glaucomatous changes in autoregulatory control of ocular hemodynamics, the glaucomatous changes in spontaneous brain activity) [37–40]. Thus, the glaucomatous IOP might be influenced by multiple factors and could not be solely explained by SC and scleral spur.

## Conclusions

Using SS-OCT, we found that the scleral spur was significantly shorter in POAG eyes compared with healthy eyes in vivo. And the scleral spur length has a good diagnostic capability in discriminating POAG eyes from healthy eyes. Thus, the scleral spur length could be a novel biomarker for POAG evaluation clinically. Moreover, the SC area was significantly associated with the scleral spur length in both POAG and healthy groups, suggesting the important role of scleral spur in the maintenance of SC morphology.
